# Mechanical properties of orthodontic wires derived by instrumented indentation testing (IIT) according to ISO 14577

**DOI:** 10.1186/s40510-015-0091-z

**Published:** 2015-06-19

**Authors:** Spiros Zinelis, Youssef S Al Jabbari, Marianna Gaintantzopoulou, George Eliades, Theodore Eliades

**Affiliations:** Department of Biomaterials, School of Dentistry, National and Kapodistrian University of Athens, Thivon 2 str., Goudi 11527 Athens, Greece; Dental Biomaterials Research and Development Chair, King Saud University, Riyadh, Saudi Arabia; Prosthetic Dental Sciences Department, College of Dentistry, King Saud University, P.O. Box 60169, Riyadh, 11545 Saudi Arabia; Department of General & Specialist Dental Practice, College of Dentistry, University of Sharjah, Sharjah, United Arab Emirates; Department of Orthodontics and Paediatric Dentistry, Center of Dental Medicine, University of Zurich, Plattenstrasse 11, CH-8032 Zurich, Switzerland

## Abstract

**Background:**

The aim of this study was the characterization of mechanical properties of representative types of orthodontic wires employing instrumented indentation testing (IIT) according to ISO 14577.

**Methods:**

Segments were cut from ten wires. The first six are made of stainless steel (SS), two are made of Ni-Ti, and the last two are made of titanium molybdenum alloys (TMA). Then, the Martens hardness (HM), the Vickers hardness (HV_IT_) based on indentation hardness (*H*_IT_), the indentation modulus (*E*_IT_), the ratio of elastic to total work (*η*_IT_), and the traditional Vickers hardness (HV_1_) were measured by IIT. The results were statistically analyzed by one-way ANOVA followed by Student-Newman-Keuls (SNK) test at *a* = 0.05. The HV_IT_ and HV_1_ data were analyzed by paired *t* test (*a* = 0.05).

**Results:**

SS wires showed the highest hardness followed by TMA and Ni-Ti alloys. However, all wires showed significantly lower HV_IT_ compared to corresponding HV_1_, a finding probably appended to elastic recovery around the indentation. *E*_IT_ for all wires tested was determined much lower than the nominal values of the corresponding alloys due to the implication of residual stress field at the slope of unloading curve. Elastic to total work ratio was ranged from 45.8 to 64.4 % which is higher than that expected for ductile alloys (<30 %).

**Conclusions:**

The products tested illustrated significant differences in their mechanical properties. Although IIT provides reliable data for hardness and elastic index of materials tested, the intense residual stress field developed during the manufacturing process significantly affects the determination of modulus of elasticity.

## Background

Mechanical properties of orthodontic wires are of paramount importance as they are deeply implicated in the efficacy of orthodontic therapy [1, 2]. Fundamental mechanical properties such as modulus of elasticity, yield strength, fracture strength, and others are mostly evaluated by tensile, bending, and torsion testings [3–5]. Recently, the instrumented indentation testing (IIT) has been adopted by the International Organization for Standardization (ISO) as an alternative methodology for testing a vast spectrum of mechanical properties such as hardness, modulus of elasticity, creep, relaxation, and more [6, 3]. The method is based on monitoring the force-indentation depth (*h*) of a specimen after loading with a standardized hardness indenter (i.e., Vickers, Bercovich, Knoop). Having a constant monitoring of force and indentation depth, the Martens hardness is determined by indentation depth under working load, minimizing the interference of optical and visco-elastic properties of the material on the diagonal length of indentations. The optical properties are associated with the difficulty to accurately determine the diagonal length in transparent material (i.e., plastic brackets) while the visco-elastic properties are associated with time-dependent properties of the material and more important the rebound of the material around indentation after load removal. This property has been initially named universal hardness, but now, the term Martens hardness is widely accepted [6]. The IIT method is fully automated and provides the advantage that small and irregular samples (such as dental devices) can be tested as final products bypassing the demand for standard specimens such as rectangular beams, dumbbells, etc. The principle, the mathematical formulas, and the properties tested are thoroughly presented in ISO 14577-1 [6] specification, where testing is classified in macro-range (2 N ≤ *F* ≤ 30 kN), micro-range (*F* ≤ 2 N, *h* > 0.2 μm), and nano-range (*h* ≤ 0.2 μm).

In the relevant literature, there are a few studies available on the mechanical properties of orthodontic wires employing nano-range conditions, a technique commonly referred as nano-indentation [7, 3, 8, 9]. Surprisingly, the hardness and modulus of elasticity, as determined by IIT, were significantly different from the results given by conventional tensile, torsion, and hardness testing [3]. This finding can be attributed to two major reasons: (a) the results of nano-indentation are dependent on the loading conditions, tending to increase from lower (2 mN) to higher loading conditions (100 mN), possibly attributed to the contribution of the native oxide film as the indentation depth is superficial, within a few hundreds of nanometer from the outer surface [8]. Such shallow indentation depths characterize more the surface and near surface properties rather than the bulk ones [7]; (b) the shallow indentation depths are strongly affected by specimen roughness, and thus, the accuracy of experimental results are sensitive to the specimen roughness state [10].

However, these limitations and concerns can be overwhelmed by employing macro-scale loading conditions. An additional advantage is that Vickers hardness based on force-indentation depth and optical measurement can be measured on the same indentation. This simplifies the comparison between the techniques rather than transform the results of hardness derived by nano-indentation hardness with Bercovich indenter to Vickers through mathematical formulas [6] and then compare with Vickers data in macro-scale [3].

Therefore, the aim of this study was the determination of the traditional Vickers hardness (HV) along with the Vickers hardness (HV_IT_), indentation modulus (*E*_IT_), and elastic to total work ratio (*η*_IT_) provided by the force-indentation depth curve. The null hypothesis is that the aforementioned properties will be different among representative types of alloys tested.

## Methods

Table 1 presents the orthodontic wires included in this study along with their commercial names, code, cross section geometry, manufacturer, and alloy type. The wires were cut into 15-mm segments employing a low-speed oil-cooled diamond saw (IsoMet, Buehler, Lake Bluff, Il), and the segments were embedded longitudinally in an epoxy resin (EpoFix, Struers, Ballerup, Denmark). Then, the specimens were metallographically ground and polished up to 1-μm alumina slurry in a grinding/polishing machine (EcoMet III, Buehler) and ultrasonically cleaned for 10 min in a water bath.

**Table 1:** Commercial names, code, cross section geometry, size, type, manufacturer, and alloy type of orthodontic wires tested in this study

Product/Code	Cross section/size	Type	Manufacturer	Alloy
A.J. Wilcock Australian wire/AJW	C, 0.018 in.^a^	S^c^	G & H Wire Company, Franklin, IN	300 series
TruForce SS/TRF	C, 0.018 in.	A^d^	Ortho Technology, TruForce, Tampa, FL	AISI 304
Penta-One wire/POW	Multistrand, 0.0155 in.	S	Masel Ortho Organizers Inc., Carlsbad, CA	AISI 304
SS arch wires/SAW	R, 0.017 × 0.025 in.^b^	S	Highland Metals Inc., San Jose, CA	AISI 304
Remanium/REM	C, 0.0155 in.	A	Dentaurum, Ispringen, Germany	AISI 304
Nominium/NOM	C, 0.0155 in.	A	Dentaurum	Ni-free SS
Superelastic regular force/RFR	R, 0.018 × 0.025 in.	A	Highland Metals Inc.	Ni-Ti
Superelastic regular force/RFC	C, 0.018 in.	A	Highland Metals Inc.	Ni-Ti
Beta blue arches/BBA	R, 0.017 × 0.025 in.	A	Highland Metals Inc.	TMA
Respond/RES	Multistrand, 0.0195 in.	S	Ormco Corporation, Glendora, CA, USA	TMA

^a^Circular

^b^Rectangular

^c^Straightened wire

^d^Arch wire

**Fig. 1:**
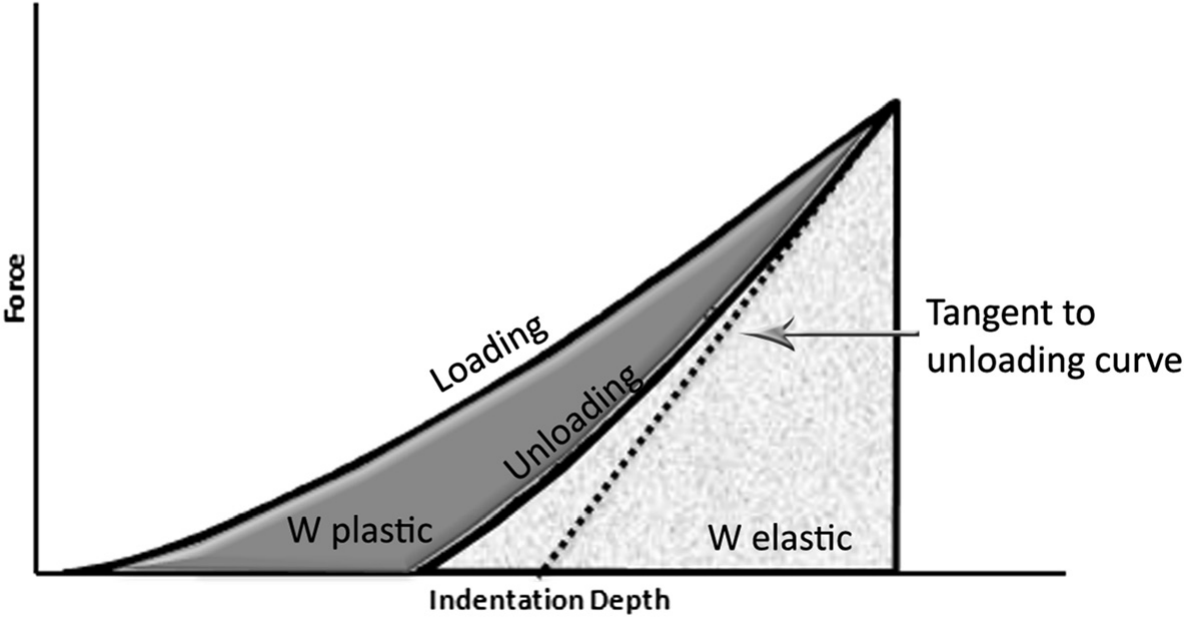
Representative loading-unloading curve obtained by instrumented indentation testing (IIT). The elastic and plastic works were highlighted by different shadings of corresponding areas while tangent to unloading curve is used for the characterization of the indentation modulus (*E*_IT_)

IIT measurements were carried out employing a universal hardness-testing machine ZHU0.2/Z2.5 (Zwick Roell, Ulm, Germany). Force-indentation depth curves were monitored applying 9.8 N with a 15-s dwell time by a Vickers indenter. Three readings were taken from the center of each specimen, and the mean value was used as representative of the specimen (*n* = 10 per product). All force-indentation depth curves were recorded, and the indentation hardness (*H*_IT_), indentation modulus (*E*_IT_), and percentage of the elastic part of indentation work (*η*_ΙΤ_), also known as elastic index, were determined according to the ISO 14577-1 specification. Finally, the Vickers hardness (HV_1_) was measured based on the diagonal of the indentation at ×10 nominal magnification. Indentation hardness (*H*_IT_) is given by the equation:

$$ {H}_{\mathrm{IT}}=\frac{F_{\max }}{A_{\mathrm{p}}} $$

where *F*_max_ is the maximum applied force and *A*_p_ is the projected (cross-sectional) area of contact between the test piece and the indenter. This can be correlated to Vickers hardness HV_IT_ by the formula HV_IT_ = 0.0945*H_IT_ provided by the ISO 14577-1 specification [6]. Martens hardness is determined by the ratio:

$$ \mathrm{H}\mathrm{M}=\frac{F}{26.43*{h}^2} $$

where *F* and *h* stand for test force and indentation depth under test force, respectively. Indentation modulus (*E*_IT_) was calculated by the following formula:

$$ {E}_{\mathrm{IT}} = \frac{1-{\left({v}_{\mathrm{s}}\right)}^2}{\frac{1}{E_{\mathrm{r}}}-\frac{1-{\left({v}_{\mathrm{i}}\right)}^2}{E_{\mathrm{i}}}} $$

where *ν*_s_ is the Poisson’s ratio of sample and *v*_i_ (0.07) the Poisson’s ratio of the indenter. The Poisson’s ratio values were set at 0.29 for stainless steel (SS) alloys, 0.3 for Ni-Ti, and 0.31 for titanium molybdenum alloys (TMA) [11]. The term *E*_i_ stands for the modulus of the indenter (1140 GPa) while *E*_r_ is the reduced modulus given by the formula:

$$ {E}_{\mathrm{r}} = \frac{\sqrt{\pi }}{2C\sqrt{A_{\mathrm{p}}}} $$

where *C* denotes the compliance of the contact and is determined by the slope of dh/dF between 95 and 60 % of *F*_max_, and thus, the steeper (more vertical) the unloading curve the higher the *E*_IT_. Finally, *η*_IT_ is given by the equation:

$$ {\eta}_{\mathrm{IT}}=\frac{W_{\mathrm{elast}}}{W_{\mathrm{total}}}*100\% $$

where *W*_elast_ is the area under the unloading curve, *W*_plast_ the area between the loading and unloading curves, and *W*_total_ the sum of elastic and plastic works determined by the total area below the loading curve (Fig. 1). All indents were made at the center of cross section, and they were located more than 2.5 indentation diameter from the edge of the specimen according to the ASTM E384 guidelines [12].

The results of HM, HV_IT_, HV_1_, *E*_IT_, and *η*_ΙΤ_ were statistically analyzed by one-way ANOVA employing the material as a discriminating variable. Significant differences among groups were allocated by post hoc Student-Newman-Keuls (SNK) multiple comparison analysis at *a* = 0.05. Paired *t* test was used to compare the Vickers hardness between the indentation and traditional testings (*a* = 0.05).

## Results

**Fig. 2:**
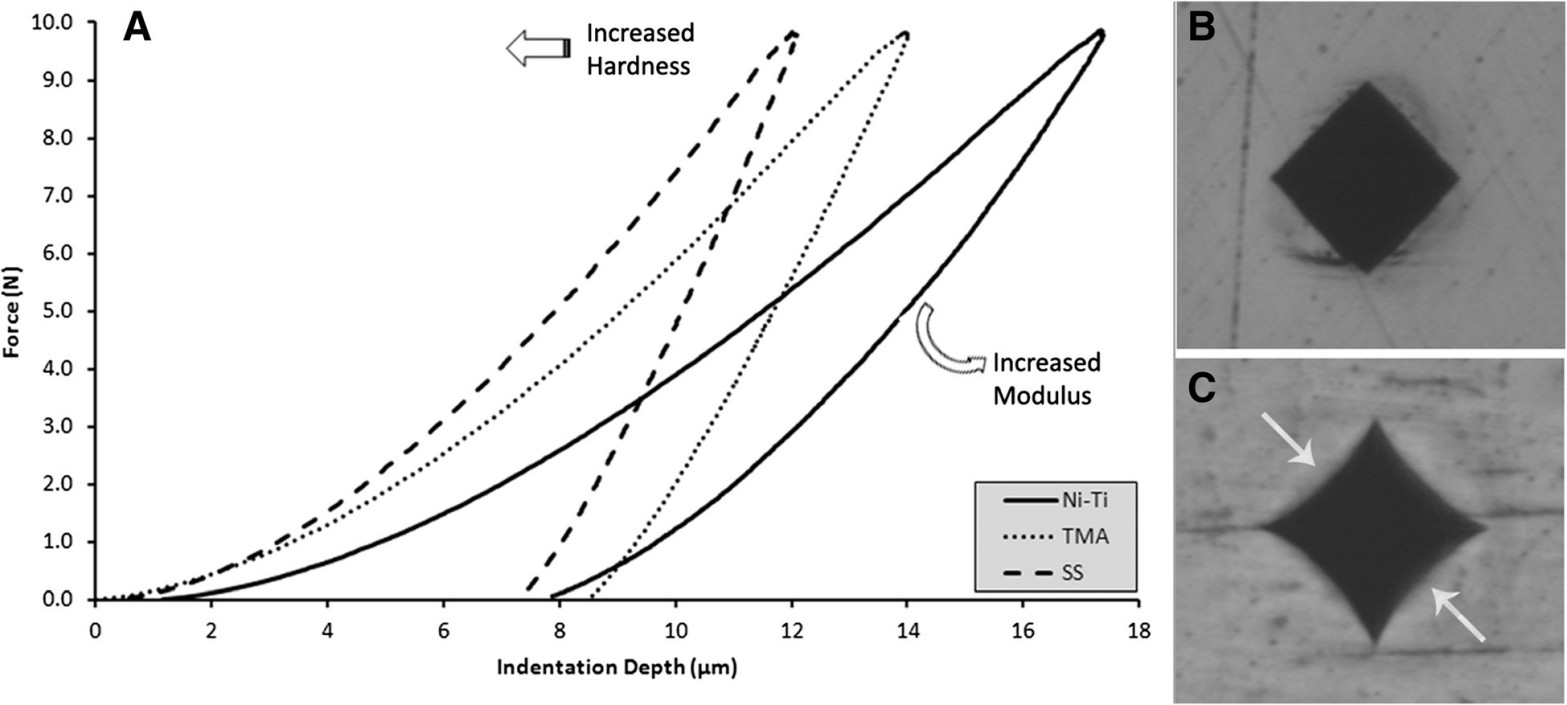
**a** Representative force-indentation depth curves of SS, Ni-Ti, and TMA wires. The *left peak shifting* denotes increase in hardness. The steeper the unloading curve the higher the indentation modulus (*E*_IT_). The Ni-Ti unloading curve changes to a smaller angle slope as the load returns to 0, indicating a noticeable elastic material recovery. **b** Representative Vickers indent of SS and TMA wires, without a noticeable elastic recovery. **c** Vickers indentation of a Ni-Ti wire with curved sides (the direction of elastic recovery is pointed by the *arrows*) indicating extensive elastic recovery of the alloy around the tip

Figure 2a demonstrates representative force-indentation depth curves of different types of alloys tested. SS wires depicted shallower indentation depth indicating higher hardness, steeper unloading, and higher *E*_IT_ compared to Ni-Ti and TMA wires. Figure 2b illustrates representative indentations for SS and TMA wires without a noticeable elastic recovery. However, the indentation of the Ni-Ti alloy showed curved sides, indicative of elastic recovery around the tip. This elastic rebound is also shown in the force-indentation depth curve as a change in the unloading curve slope at forces below 1 N, implying a rapid decrease in indentation depth.

HV_IT_ were found significantly lower compared to HV_1_ between different Vickers methods for all materials tested (Table 2). The SS alloys showed significantly higher *H*_IT_ and HV_1_ compared to Ni-Ti and TMA alloys apart from RES which showed an intermediate value. The same trend was found for HM and *E*_IT_ with SS showing the highest values and the Ni-Ti the lowest ones while both TMA wires showed intermediate results. Elastic to total work ratio *η*_IT_ cannot be sorted according to material type showing a rather random distribution among materials tested.

**Table 2:** Mean values and standard deviation of HV_IT_, H_1_, HM, E_IT_, and η_IT_ for all the products tested

Material	Vickers hardness, HV_IT_(=0.0945 *H*_IT_) HV_1_		HM, (N/mm^2^)	*E*_ΙΤ,_ (GPa)	*η*_ΙΤ_, (%)
AJW	475(36)^1, a^	545(8)^1,2, b^	2558(165)^1^	42.5(2.1)^1^	57.3(2.9)^1,2^
TRF	462(18)^1,2, a^	529(11)^2, b^	2656(76)^1^	49.7(0.9)^2^	47.0(0.7)^3^
POW	476(26)^1, a^	535(4)^1,2, b^	2211(85)^2^	34.3(1.3)^3^	58.2(0.4)^2,4^
SAW	430(6)^2,3, a^	528(7)^2, b^	2282(27)^2^	39.1(0.5)^4^	51.3(0.5)^5^
REM	429(13)^2,3, a^	601(3)^3, b^	2123(56)^2^	33.8(0.8)^5^	55.3(0.6)^1^
NOM	419(10)^3, a^	528(6)^1,2, b^	1831(36)^3^	27.1(0.5)^6^	64.4(0.9)^6^
RFR	304(2)^4, a^	333(5)^4, b^	1548(11)^4^	25.0(0.3)^6^	64.1(0.9)^6^
RFC	249(9)^5, a^	325(15)^4, b^	1287(32)^5^	21.0(0.4)^7^	59.8(0.3)^4^
BBA	309(14)^4, a^	330(9)^4, b^	1818(58)^3^	34.2(0.6)^3^	45.8(1.4)^3^
RES	397(22)^3, a^	488(21)^5, b^	2033(29)^2^	33.2(1.1)^5^	51.8(1.6)^5^

Same numerical superscripts denote mean values without statistical significant differences among materials (*P* > 0.05). Same alphabetic superscripts illustrate no significant differences (*P* > 0.05) between HV_IT_ and HV_1_

## Discussion

The null hypothesis must be accepted as the material tested showed significant differences in tested properties. The products tested represent many of the material and geometrical parameters of contemporary orthodontic wires. Four wires (TRF, POW, SAW and REM) are made of AISI 304 SS alloy with nominal composition (wt%): Fe: Balance, Cr: 18–20, Ni: 8.0–10.5, Mn < 2.0, Si < 1.0, *P* < 0.045, S < 0.03, and C < 0.08 [11]. Three products (TRF, POW, and SAW) are delivered as preformed arches, while POW is a multistrand wire. The alloy type of A.J. Wilcock Australian wire (AJW) is not given by the manufacturer, but previous reports advocate that it belongs to 300 series SS, with Cr content in the range of 17–25 % and Ni content in the range of 8–12 % [13]. AJW is delivered in a spooled form with an increasing resilience (regular to supreme grades), but only the regular grade was included in this study [13]. NOM is a Ni-free alloy with nominal composition (wt%) Fe: Balance, Cr: 16.0–20.0, Ni ≤ 0.2, Mo :1.8–2.5, Mn :16.0–20.0, Si ≤ 1.0, *P* ≤ 0.05, S ≤ 0.05, C ≤ 0.1, V ≤ 0.2, and N: 0.7–1.0. This alloy is delivered with the 1.4456 EN/DIN numerical designation corresponding to the S 310 50 in UNS designation, but without a code in the AISI USA system [14].

There is no data for HM in dental literature, but the results of Vickers hardness are in agreement with previous findings with the SS wires demonstrating the highest hardness (484 ~ 600 HV [15, 16, 3]) compared to other alloys. Ni-Ti (240 ~ 438 HV [7, 3, 15, 9, 5]) and TMA (292 ~ 377 HV [3, 15, 17, 5]) showed lower values with overlapping ranges. However, the recorded values for orthodontic wires are much higher than the nominal values of AISI 304 (210 HV [18]) and Ni-Ti (200 HV [19]) alloys in annealed state, due to the extensive cold working during the manufacturing process [5], the extent of which remains unknown as the thermomechanical treatment of each product is considered proprietary [5]. The variations in thermomechanical treatment among the products may explain the different hardness values of wires sharing the same elemental compositions.

Interestingly, all materials showed significantly lower HV_IT_ compared to HV_1_ with difference in mean values ranging from 21 up to 172 Vickers. This finding shows that the results between the two methods are not comparable, although both methods measure the same material property. The accuracy of traditional Vickers hardness measurement is influenced mainly by the resolution of the optical system, the operator’s perception, the variation of hardness with load, (a phenomenon commonly known as indentation size effect), and most importantly, by the elastic recovery of the material around indention after load removal [20]. All these parameters may overestimate or underestimate the final outcome, apart from the elastic recovery, which constantly overestimates the measurement as it decreases the diagonal length. Contrary, indentation hardness testing is free of all these interferences. Ni-Ti showed higher elastic recovery during unloading (Fig. 2), but this behavior should not be confused with shape memory and pseudoelasticity of Ni-Ti system as these properties cannot be activated in cold work state as it has been indicated for orthodontic wires and endodontic files made of Ni-Ti alloy [21–23]. Nevertheless, although fully automated, the results of indentation hardness are still influenced by some experimental parameters (i.e., approach speed of the indenter, test force or indentation depth control mode, speed of application of the test force) and thus, small variations among IIT results are anticipated.

The *E*_IT_ values were found according to the expected classification with SS showing the highest values followed by TMA and Ni-Ti wires. Yet, the results are much lower than the nominal values of orthodontic wires (168 ~ 226 GPa for SS, 57 ~ 86 GPa for TMA, and 30 ~ 44 GPa for Ni-Ti [1]). However, this inconsistency cannot be appended to experimental conditions as loading conditions applied according to ISO 14577 [6] and indentations were substantial enough distance from the edge of the sample according to the ASTM E384 guidelines [12]. As described in Fig. 1, *E*_IT_ is measured by the slope of the unloading curve and the steeper the slope the higher the modulus. The unloading cycle starts at maximum depth after the application of preselected load. At this point, the external force of the device is set to 0 but the indenter moves backwards due to the elastic rebound of the material. The device is capable of monitoring the exerted force and indentation depth simultaneously drawing the unloading curve. However, the slope of this curve is strongly affected by the presence of residual stresses, overestimating and underestimating the *E*_IT_ values in compressive and tensile residual stresses, respectively [24]. Taking advantage of this phenomenon, a certain methodology has been developed for the exact estimation of residual stresses using stress-free samples as reference [24]. The aforementioned comments explain that the estimation of reliable values for *E*_IT_ requires stress-free samples. This might be also the explanation that the results of previous studies with nano-indentation although demonstrated closer results to nominal values of orthodontic alloys for SS (150 ~ 229 GPa [3, 7]), Ni-Ti (60 ~ 69 GPa [9, 7, 3]) and TMA alloys (68 [3] GPa) failed to match with the tensile results when the same alloy is tested by both methods [3]. The necessity for stress-free samples is not clearly presented as a prerequisite for the proper estimation of modulus in relevant documents, and thus, researcher must be aware of this limitation to avoid the presentation of fault and misleading data.

The force-indentation curve (Fig. 1) provides also information for the total work of indentation *W*_total_, which is divided in elastic *W*_elast_ and plastic works *W*_plast_. However, as the absolute values are dependent on the applied load, it is preferred to make comparisons based on normalized quantities, like the elastic index *η*_ΙΤ_ which is independent of load, [25]. In the present study, the elastic indices showed much higher than the expected values for ductile alloys (<30 % [26]). Especially for Ni-Ti alloys, this is an additional evidence that they are not in fully annealed form and thus are not capable of showing shape memory and superelastic properties.

The clinical implication of hardness data is associated with the arch wire itself and the matching with mechanical properties of bracket. Since hardness is an indication for the material resistance in plastic deformation, the higher the hardness of the alloy the higher the resistance to plastic deformation. A recent study has experimentally verified that SS has better wear resistance (followed by TMA with intermediate and Ni-Ti with the worst) against both 316 SS and Ti-6Al-4V bracket alloys [16]. Therefore, to minimize the wear between wire and bracket, materials with similar hardness must be used [16] and thus, IIT methodology and especially HM might be used to provide a more accurate image of hardness of orthodontic materials. However, wear and surface phenomena between brackets and wires are much more complex and cannot be simply explained by a bulk material property such as hardness itself. IIT is a modern, standardized, and fully automated experimental methodology and should be used further to deepen our knowledge on the mechanical behavior of orthodontic materials.

## Conclusions

- IIT provided lower Vickers hardness data compared to traditional Vickers testing for all types of wires tested.
- IIT can provide reliable data for mechanical properties of materials tested, but the residual stress field of orthodontic wires seriously interfere with the estimation of indentation modulus.
